# Public Hospitals in China: Is There a Variation in Patient Experience with Inpatient Care

**DOI:** 10.3390/ijerph16020193

**Published:** 2019-01-11

**Authors:** Wenhua Wang, Ekaterina (Katya) Loban, Emilie Dionne

**Affiliations:** Department of Family Medicine, McGill University, Montreal, Quebec H3T 1M5, Canada; ekaterina.loban@mail.mcgill.ca (E.K.L.); emilie.dionne@mail.mcgill.ca (E.D.)

**Keywords:** public hospital, patient centeredness, patient responsiveness, patient experience, inpatient care, disparities, equity

## Abstract

In China, public hospitals are the main provider of inpatient service. The Chinese public hospital reform has recently shifted towards health care organizations and delivery to improve health care quality. This study analyzes the variation of one of the dimensions of health care quality, patient-centeredness, among inpatients with different socioeconomic status and geographical residency in China. 1471 respondents who received inpatient care in public hospitals were included in our analysis. Patient-centeredness performance was assessed on the dimensions of Communication, Autonomy, Dignity, and Confidentiality. Variations of inpatient experience were estimated using binary logistic regression models according to: residency, region, age, gender, education, income quintile, self-rated health, and number of hospital admissions. Our results indicate that older patients, and patients living in rural areas and Eastern China are more likely to report positive experience of their public hospital stay according to the care aspects of Dignity, Communication, Confidentiality and Autonomy. However, there remains a gap between China and other countries in relation to inpatient experience. Noticeable disparities in inpatient experience also persist between different geographical regions in China. These variations of patient experience pose a challenge that China’s health policy makers would need to consider in their future reform efforts.

## 1. Introduction

In China, public hospitals account for the majority of hospital beds and medical staff in the health care system. In 2015, out of all hospital admissions in China, the inpatient admission in public hospitals comprised 85.3% [1]. Public hospital reform in China commenced in 2009 and is still ongoing, with the majority of the initial reform efforts focusing on hospital governance, financing, payments and incentives [2]. For example, some cities reorganized the responsibilities and powers of government departments by limiting the policy-making power of the Department of Health and creating a new agency to manage public hospitals [2]. In addition, some regions altered financing policies by increasing government subsidies for basic construction, equipment purchasing, and pensions for retirees [2]. Furthermore, most cities have established a compensation system to pay staff a basic salary plus a performance-based bonus [2]. A recent evaluation shows that this important systemic reform has reduced the medical care cost per inpatient admission by 15.4% [3]. This was largely accomplished through a decrease in drug expenditures per inpatient admission of approximately 53% [3]. More recently, the Chinese health care reform emphasis has shifted from health care organizations and delivery to health care quality improvement [4]. There is a growing agreement in China that deeper reforms are required to improve hospital performance in quality of care, especially patient experience and satisfaction [5,6,7,8,9].

One of the six dimensions of health care quality, person-centeredness, is defined by the National Academy of Medicine as “providing care that is respectful of and responsive to individual preferences, needs, and values and ensuring that people’s values guide all clinical decisions.” [10] There is consensus that patients are the best source of information for evaluating the care aspects of person-centeredness, such as communication, respect, autonomy, and privacy [11,12,13,14]. To measure patient experience performance, instruments such as the Picker Patient Experience Questionnaire [15,16], and the Hospital Consumer Assessment of Healthcare Providers and System [17,18] have been developed and validated in the UK and the USA, respectively. Evidence demonstrates that good patient experience is associated with higher levels of adherence to recommended prevention and treatment plans, better clinical effectiveness and outcomes, better patient safety within hospitals, and lower health care utilization [19,20].

Previous studies in China highlight that interpersonal care quality is a strong predictor of the overall rating of inpatient care [21,22,23]. Patient-centered care aspects have been most frequently mentioned in a recent qualitative study exploring patient priorities in China [24]. For example, good communication skills are appreciated by many patients. Respect for patients’ views and protection of patients’ privacy are also mentioned by some participants as an important facet of the quality of the health care they received. The majority of patients express a wish to be involved in decision making such as discussing their treatment protocols with doctors [24]. However, information about hospital performance in these care aspects prioritized by patients in China remains sparse.

The World Health Organization (WHO) Study on global AGEing and adult health (SAGE) is a longitudinal study with nationally representative samples in six low- and middle-income countries, including China [25]. The WHO SAGE survey includes information on key patient-centered care aspects and patients’ overall satisfaction with their recent hospitalization. Using the data from the WHO SAGE for China and the Minority Health and Health Disparities Research Framework from the National Institute of Minority Health and Health Disparities, this paper explores patient-centeredness performance rated by patients on the dimensions of Communication, Autonomy, Dignity, and Confidentiality, in order to analyze performance variation among patients with different socioeconomic status and living in different geographical locations in China. 

## 2. Methods

### 2.1. Data Source 

The WHO SAGE study includes nationally representative samples of persons aged 50 years and older in China, Ghana, India, Mexico, the Russian Federation and South Africa, with comparison samples of younger adults aged 18–49 years in each country [25]. Based on a multistage cluster sampling design, face-to-face interviews were conducted by trained interviewers to collect information on sociodemographics, health risk factors and chronic conditions, health service utilization and patient responsiveness using a standardized instrument. SAGE Wave 1 in China was completed in 2010 and included 1636 respondents aged 18–49 and 13,177 respondents aged 50 years and above in eight provinces in China. The overall response rate was over 98% [26,27,28]. 1574 of all respondents from China reported that they had at least one overnight stay in a hospital or other health facility in the past 12 months; 1471 of which were in public hospitals. These 1471 respondents who received inpatient care in public hospitals were included in our analysis.

### 2.2. Measurement of Patient Experience

Four items from the WHO Patient Responsiveness Survey measure four care aspects of patient-centeredness: Communication, Autonomy, Dignity, and Confidentiality [29,30]. Patients were asked to rate each of the following care aspects of their most recent hospital stay: (1) Communication: how clearly health care providers explained things to you? (2) Autonomy: your experience of being involved in making decisions for your treatment? (3) Dignity: your experience of being treated respectfully? and (4) Confidentiality: the way the health services ensured that you could talk privately to providers? A five-point Likert response scale ranging from 1 = very good to 5 = very bad was used. The answers were dichotomized into “good” (including “very good” and “good”) and “bad” (including “moderate”, “bad” and “very bad”) for further analysis.

### 2.3. Individual Characteristics

Research from other countries demonstrates that age and self-rated health status typically have the strongest and most consistent associations with inpatient experience [31]. However, there are inconsistent results in relation to the association with other characteristics, such as sex, education, income, and rating of hospital care [31]. Research conducted into the United States’ hospital care has found that there were large regional variations in patient experiences with their care [32], and most variation in ratings and reports about health care providers was attributable to geographic factors, particularly the geographical variations in medical practice styles [33].

Based on previous research [31,32,33], eight factors were included in this study. The two geographic factors included: residency (urban/rural), and region (Eastern, Central, and Western China). The six individual characteristics were as follows: age, gender, education, income quintile, self-rated health, and number of hospital admissions. Age was dichotomized into 18–59 years versus 60-plus years old. Education included four categories: less than primary school, primary school, secondary school, and high school or above. The household income quintiles were based on the possession of a set of household assets and a number of dwelling characteristics with Q1 representing the poorest household category and Q5 representing the richest household category [26,27,28]. The correlation coefficients between age, education, and income quintiles range from 0.005 to 0.308, which suggests that they can be included in the multiple regression model together. Self-rated health was categorized into “good” (including “very good” and “good”), “moderate”, and “bad” (including “bad” and “very bad”) [28]. The number of inpatient admissions over the last 12 months was categorized into two groups: 1 time and 2 times or more.

### 2.4. Statistical Analysis

Statistical analysis was performed using SPSS 22.0 (SPSS Inc., Chicago, IL, USA). Missing values were excluded list wise. First, we introduced the characteristics of 1471 patients admitted to public hospitals during the last 12 months. Second, percentages of patients reporting “good” care in Communication, Autonomy, Dignity, and Confidentiality were described by sub-groups of each patient characteristic; a chi-square test was subsequently used to test the differences of percentages between sub-groups of each patient characteristic. Third, separate binary logistic regression models were used to examine the association between individual characteristics and their rating of hospital care. 

For the four items measuring Communication, Autonomy, Dignity, and Confidentiality, the percent of missing values was 7.5%. Considering the high percent of missing values on the four items, we conducted sensitivity analysis after replacing the missing values of the four items to ensure the stability of our results. Based on previous WHO health system responsiveness analysis results, four variables including age, income, education and self-rated health were selected as match variables to complete missing data using the maximum likelihood estimation method in LISREL 9.1. After replacing the missing values of the four items using maximum likelihood estimation, there was no change in our conclusions.

### 2.5. Research Ethics Approval

SAGE has been approved by the WHO’s Ethical Review Committee. In addition, each partner organization implementing SAGE obtained ethical clearance through their respective review bodies. Written informed consent was obtained from all study participants. [25]

## 3. Results

Sociodemographic characteristics and self-rated health status of 14,813 total respondents are reported in Table 1, along with the characteristics of 1471 respondents receiving inpatient service in public hospitals over the last 12 month. For inpatients in public hospitals, the average age was 64.1 years old, 52% were female, 21.0% were illiterate, less than 20% reported good health status, and 13.1% had multiple admissions over the last 12 months (Table 1). 

### 3.1. Communication

Overall, Communication for the most recent hospitalization was rated as “good” by 77.7% of the respondents. We noticed an important geographical variation in patient perceived Communication. The percentage of patients reporting good Communication was higher in rural areas compared to urban areas (82.6% vs. 73.3%, respectively) and higher in the Eastern region compared to Central and Western regions (80.3% vs. 76.3% vs. 73.8%, respectively). Patients with lower education attainment level and good health status were more likely to report good Communication (Table 2).

The multiple regression analysis confirmed that patients in rural areas were more likely to report good Communication (vs. urban areas; OR: 1.97; 95% CI: 1.33–2.93) while patients in non-Eastern regions were less likely to report good Communication. The association between education and self-rated health and Communication rating disappeared, while the association between age and Communication became apparent. Finally, older patients were more likely to report good Communication than younger patients (OR: 1.81; 95% CI: 1.29–2.54) (Table 3).

### 3.2. Autonomy

Overall, Autonomy for the most recent hospitalization was rated as “good” by 71.6% of the respondents. Here too we noted an important geographical variation in patient perceived Autonomy. The percentage of patients reporting good Autonomy was higher in rural areas than urban areas (76.2% vs. 67.5%, respectively) and in the Eastern region than the Central and Western regions (78.3% vs. 64.0% vs. 64.9%, respectively). Patients with good health status were more likely to report good Autonomy; however, patients with multiple hospital admissions over the last 12 months were less likely to report good Autonomy compared with patients with only one admission (64.4% vs. 72.8%) (Table 2). 

The multiple regression analysis confirmed that patients in rural areas were more likely to report good Autonomy (vs. urban; OR: 2.18; 95% CI: 1.51–3.15) while those in non-Eastern regions were less likely to report good Autonomy. The association between self-rated health and Autonomy rating disappeared, while the association between age and Autonomy rating became apparent. Older patients were more likely to report good Autonomy than younger patients (OR: 1.51; 95% CI: 1.10–2.07). Finally, patients with multiple hospital admissions were less likely to report good Autonomy (vs. one admission; OR: 0.60; 95% CI: 0.41–0.90) (Table 3).

### 3.3. Dignity

Dignity for the most recent hospitalization was rated as “good” by 81.0% of the respondents. Our results show an important geographic variation in patient perceived Dignity. The percentage of patients reporting good Dignity was higher in rural areas than in urban areas (85.0% vs. 77.4%, respectively) and also higher for patients in the Eastern region than the Central and Western regions (83.9% vs. 77.1% vs. 78.7%, respectively). Patients with lower education attainment levels and good health status were more likely to report good Dignity than others (Table 2).

The multiple regression analysis confirmed that patients in rural areas were more likely to report good Dignity (vs. urban; OR: 1.93; 95% CI: 1.26–2.95) while patients in non-Eastern regions were more likely to report lower Dignity. The association of education and self-rated health with high rating of Dignity disappeared, while the association between age and Dignity became apparent. Older patients were more likely to report good Dignity than younger patients (OR: 1.88; 95% CI: 1.31–2.70) (Table 3).

### 3.4. Confidentiality 

Overall, Confidentiality during the most recent hospitalization was rated as “good” by 74.9% of the respondents. A large geographical variation in patient perceived Confidentiality remains in relation to this area as well. The percentage of patients reporting good Confidentiality was higher in rural areas than in urban areas (77.9% vs. 72.1%, respectively) and higher for patients in the Eastern region than in the Central and Western regions (79.9% vs. 73.3% vs. 66.5%, respectively). Patients with good self-rated health status were more likely to report good Confidentiality than those with low health status (Table 2).

The multiple regression analysis confirmed that patients in rural areas were more likely to report good Confidentiality than those living in an urban setting (OR: 1.81; 95% CI: 1.24–2.63). Patients in the Eastern region were less likely to report bad Confidentiality than those living in the Central region (OR: 0.54; 95% CI: 0.36–0.82) or the Western region (OR: 0.35; 95% CI: 0.23–0.52). The association between self-rated health and Confidentiality rating disappeared, while the association between age and Confidentiality rating was revealed. Older patients were more likely to report good Confidentiality than younger patients (OR: 1.67; 95% CI: 1.20–2.31) (Table 3).

## 4. Discussion

In relation to the four interpersonal care aspects examined in inpatient service provision, Chinese public hospitals performed best in Dignity (81% of respondents rating it as “good”), followed by Communication (78% of respondents rating it as “good”), Confidentiality (75% of respondents rating it as “good”), and, finally, Autonomy (72% of respondents rating it as “good”). Studies conducted in South Africa, Brazil, Israel, and European countries demonstrate a similar performance ranking in the same care aspects [34,35,36,37]. Performance ranks in these aspects are also consistent with patient priorities’ ranks in these countries. A previous study looking at patient priorities of non-clinical quality of care in 41 countries demonstrated that Dignity was selected as the most important domain by most respondents, followed by Communication and Confidentiality, leaving Autonomy as the least valued domain of the four interpersonal care aspects [29].

While there is similarity between the ranking of these care aspects between China and other countries, variation exists in the actual performance. For example, China performs better in Dignity and Confidentiality than South Africa, but worse than Brazil, Israel, and some of the European countries. China lags behind Israel and some European countries in Communication and Autonomy [34,35,36,37]. 81% of inpatients report “good” or “very good” Dignity during their hospital stay in China, which is higher than South Africa (74%), but lower than Brazil (90%), Israel (90%), and a number of European countries (89%) [34,35,36,37]. This could be due to different health system features, hospital characteristics, physician qualification and professionalism, and population preference of health care. Such important variations in patient responses exist not only between developing countries and developed countries, but also within developed countries. For example, a cross-country study in Europe identified important disparities in patient responsiveness to health systems between and within European countries [38]. Previous studies suggested that in order to achieve high-quality universal health coverage and improve patient experiences, multi-level approaches were needed, such as strengthening political rights, increasing health care spending, and expanding private sector provision [39,40,41]. Further research is, however, needed to assess the effect of such interventions to improve patient experience and, subsequently, quality of care in China. Furthermore, attention to geographical differences, as well as historical and social roots of health care system set-up and provision, could provide contextual information supporting the understanding of observed disparities in patient experience of care in China and inform policy-making.

Our study also confirms that age is an important predictor of hospital experience rating in China (gender, education, income and self-rated health status are not associated with variation in patient experience of inpatient care). People over 60 years old are more likely to report a positive experience in Dignity, Communication, Confidentiality, and Autonomy than people younger than 60 years (ranging from 1.5 times to 1.9 times). This is consistent with studies conducted in other countries [31,42]. Several explanations have been proposed to explain why older people generally report more positive care experience or higher satisfaction. For example, one study reports that older people are more “stoical, mellow and accepting” than younger people [43]. Older people may also feel “more reluctant” than younger patients “to give negative judgement on their care” [44]. The study also reports that older people tend to “engender more respect and receive more responsive and positive care from their provider”. Finally, a previous study also proposes that there is a cohort effect whereby older people have lower expectations toward the health care system due to prior experiences [43,44]. Further studies are required to examine if such explanations are applicable to the Chinese population.

Chinese patients with two or more hospital admissions over the last 12 months were more likely to report negative Autonomy than others. This result is consistent with previous studies from the United States that report a negative association between the patient rating of inpatient care and readmission [45,46,47]. Our study, however, lacks data on the conditions of admission of this patient subgroup, which would confirm if these patients were readmitted to the hospital for the same condition or for different ones. In either situation, it is possible that patients with frequent health service utilization possess more in-depth information about the medical conditions they have and about potential treatment options. In such cases, patients could be more likely to have a personal preference regarding treatment or expect to be involved in decision-making, such as discussing their treatment protocols with doctors. Furthermore, although Autonomy was rated as the least important care aspect by patients in previous studies, a recent qualitative inquiry [24] into patient priorities in public hospitals in China showed that, in addition to prioritizing caring attitude, respect, and privacy, the majority of patients expected to be involved in the decision-making process [24]. Further research is, however, needed to examine the trend of population preference change in the Chinese population. Such information would support a better insight into the notions of patient involvement and shared-decision making specific to this population.

Regional variation in inpatient care experience in China is equally important. Rural patients and patients in Eastern China are more likely to report positive experience of their hospital stay in the care aspects of Communication, Autonomy, Dignity, and Privacy, than patients from other regions. Other countries have also reported similar findings with regards to urban vs. rural settings [32]. Different factors could explain this finding, such as differences in sociodemographic characteristics between people living in urban vs. rural regions. Another explanation could be that while rural regions may have fewer health care services or service options, the lower population to health providers ratio could enable patients to experience better access to providers or services and develop more enduring relationships. Conversely, fewer services or service options may contribute to patients’ lower expectations regarding health care system responsiveness in remote areas as opposed to urban settings. Further research is needed to validate these hypotheses and assess their relevance in the context of China. Geographical variations in medical resources and medical practice styles may also contribute to such variations in patient experience as style of caregiving, organizational leadership, and quality management that focus on optimizing patient experience [32,33]. Eastern China is the most developed region; it holds the most public hospitals equipped with highly qualified health professionals and sophisticated technology. This region, however, also has more public hospitals that are undergoing important reforms, of their organization structure and management practices. This likely contributes to the observed geographical variations of inpatient experience of our findings. Further research is, however, needed to validate this hypothesis. 

There are several limitations in this study. First, only one item was used to measure each care aspect of patient-centeredness: Communication, Autonomy, Dignity, and Confidentiality, which may not capture the full information regarding each aspect. Considering the population preferences and the health care system structure and operation in China, valid patient survey instruments should be developed to monitor and evaluate these interpersonal care aspects. Second, in the WHO SAGE study, the types of hospitals or facilities were categorized as: Public hospital, Private hospital, Charity or church-run hospital, and Old person’s home or long-term care facility. However, in China, hospitals are generally classified as different levels (Level 1, 2, and 3) and different types (general or specialized). Therefore, separate analyses for public hospitals with different classification levels and types could not be conducted because of data limitations. Third, separate analyses for specific hospitalization condition could not be conducted because of data limitations. Patients with different health problems or conditions may have preferences regarding different aspects of interpersonal care quality during their hospital stay. In future research, patient perceived quality between different health conditions and between different types or levels of hospitals should be examined, which can provide more specific information in order to promote patient centered care. 

## 5. Conclusions

While China has invested in large-scale health system and performance-measurement reforms aimed at improving hospital performance in quality of care, an important gap still remains between China and other countries in relation to patient experience of inpatient care. Noticeable disparities in patient experience of inpatient care also persist between different geographical regions (urban vs. rural; Eastern vs. Central vs. Western China). These between-country and within-country variations of patient experience pose a challenge that China’s health policy makers should consider in their subsequent reform efforts. Specifically, attention to context and geographical differences could clarify why reforms have been more successful in certain regions as opposed to others. A differential approach may be warranted to address patient responsiveness in various regions in an equitable fashion. Further studies exploring population preference of health care could help to identify the needs, desires and expectations of the Chinese population in relation to the health care system and service providers, and the differences among subgroups. Lastly, the effects of characteristics of health care systems, hospitals, and health professionals on patient experience should be studied, in order to develop robust patient experience improvement strategies and interventions. 

## Figures and Tables

**Table 1:** Characteristics of total respondents and inpatients discharged from public hospitals *n* (%) ^a^.

Characteristics	Total Respondents (*n* = 14,813)	Inpatients in Any Type of Hospitals (*n* = 1574)	Inpatients in Public Hospital (*n* = 1471)
Residency			
Urban	7215 (48.7)	805 (51.1)	756 (51.4)
Rural	7598 (51.3)	769 (48.9)	715 (48.6)
Region			
Eastern	7531 (50.8)	768 (48.8)	753 (51.2)
Middle	3574 (24.1)	338 (21.5)	331 (22.5)
Western	3708 (25.0)	468 (29.7)	387 (26.3)
Gender			
Male	6887 (46.5)	747 (47.5)	705 (47.9)
Female	7924 (53.5)	827 (52.5)	766 (52.1)
Age (Mean ± SD)	60.53 ± 11.93	63.97 ± 11.33	64.12 ± 11.14
18–59	7337 (49.5)	580(36.9)	537 (36.5)
60–	7474 (50.5)	994 (63.2)	930 (63.5)
Education			
Less than primary school	2492 (21.9)	249 (21.2)	230 (21.0)
Primary school	2862 (25.1)	281 (24.0)	261 (23.8)
Secondary school	3192 (28.0)	335 (28.6)	314 (28.6)
High school or above	2856 (25.1)	307 (26.2)	292 (26.6)
Income quintile			
Poorest	2809 (19.1)	292 (18.7)	267 (18.3)
Q2	2917 (19.8)	282 (18.1)	263 (18.0)
Q3	2939 (19.9)	307 (19.7)	282 (19.3)
Q4	3045 (20.7)	360 (23.1)	347 (23.8)
Richest	3031 (20.6)	320 (20.5)	300 (20.6)
Self-Rate Health status			
Very good/Good	5378 (36.9)	286 (18.1)	267 (18.2)
Moderate	6412 (44.0)	663 (42.1)	620 (42.1)
Very bad/Bad	2778 (19.1)	624 (39.7)	584 (39.7)
Number of admissions ^b^			
1	-	-	1278 (86.9)
2 or more	-	-	193 (13.1)

^a^ The percentages were calculated after excluding missing values of each variable. ^b^ Number of admissions means the total number of inpatient admissions during the last year. “-” means “not applicable”.

**Table 2:** Percentage (%) of respondents reporting Very good/Good in each domain.

Characteristics	Communication	Autonomy	Dignity	Confidentiality
Residency				
Urban	73.3 ***	67.5 ***	77.4 ***	72.1 *
Rural	82.6	76.2	85.0	77.9
Region				
Eastern	80.3 *	78.3 ***	83.9 *	79.9 ***
Middle	76.3	64.0	77.1	73.3
Western	73.8	64.9	78.7	66.5
Gender				
Male	76.9	71.6	81.3	74.8
Female	78.5	71.7	80.8	75.0
Age				
18–59	75.2	70.9	78.6	73.1
60–	79.2	72.0	82.5	75.9
Education				
Less than primary school	86.0 **	77.1	88.8 *	80.8
Primary school	78.3	70.1	80.9	74.3
Secondary school	78.3	73.4	80.7	78.3
High school or above	72.4	69.5	78.5	71.3
Income quintile				
Poorest	75.1	67.7	78.7	68.9
Q2	82.4	75.3	83.7	79.5
Q3	78.5	71.5	83.6	76.2
Q4	78.5	71.3	81.4	75.7
Richest	74.2	72.1	78.4	73.9
Self-Rate Health status				
Very good/Good	84.4 *	81.1 ***	87.7 *	81.9 *
Moderate	75.8	70.5	78.8	73.0
Very bad/Bad	76.8	68.6	80.5	73.7
Number of admissions				
1	78.5	72.8 *	81.4	75.7
2 or more	72.9	64.4	78.7	69.7
Total	77.7	71.6	81.0	74.9

Note: Chi-square tests were used to test the differences of percentages between sub-groups of each patient characteristic; *** *p* < 0.001; ** *p* < 0.01; * *p* < 0.05.

**Table 3:** The effects of individual characteristics on patient experience using separate multiple binary logistic regression models Odd Ratios (95% CI).

Characteristics	Communication (*n* = 1011)	Autonomy (*n* = 1012)	Dignity (*n* = 1012)	Confidentiality (*n* = 1012)
Residency (ref. = urban)	1.97 *** (1.33, 2.93)	2.18 *** (1.51, 3.15)	1.93 ** (1.26, 2.95)	1.81 ** (1.24, 2.63)
Region (ref. = Eastern)				
Middle	0.59 * (0.38, 0.90)	0.37 *** (0.25, 0.55)	0.46 *** (0.30, 0.73)	0.54 ** (0.36, 0.82)
Western	0.52 ** (0.35, 0.79)	0.39 *** (0.27, 0.58)	0.49 ** (0.31, 0.77)	0.35 *** (0.23, 0.52)
Sex (ref. = male)	1.31 (0.95, 1.81)	1.15 (0.86, 1.56)	1.08 (0.76, 1.53)	1.25 (0.91, 1.70)
Age (ref. = 18–59)	1.81 *** (1.29, 2.54)	1.51 * (1.10, 2.07)	1.88 *** (1.31, 2.70)	1.67 ** (1.20, 2.31)
Education (ref. = less than primary school)				
Primary school	0.73 (0.44, 1.22)	0.92 (0.58, 1.44)	0.67 (0.39, 1.18)	0.88 (0.55, 1.40)
Secondary school	0.93 (0.56, 1.57)	1.37 (0.86, 2.17)	0.85 (0.48, 1.48)	1.32 (0.81, 2.15)
High school or above	0.67 (0.39, 1.14)	1.08 (0.67, 1.76)	0.71 (0.40, 1.27)	0.88 (0.53, 1.46)
Income (ref. = poorest)				
Q2	1.34 (0.73, 2.47)	1.36 (0.78, 2.37)	1.18 (0.61, 2.28)	1.43 (0.80, 2.54)
Q3	1.36 (0.76, 2.43)	1.41 (0.83, 2.41)	1.39 (0.74, 2.62)	1.63 (0.94, 2.82)
Q4	1.11 (0.63, 1.96)	0.87 (0.51, 1.46)	0.85 (0.46, 1.57)	1.01 (0.59, 1.72)
Richest	0.82 (0.45, 1.50)	0.85 (0.48, 1.51)	0.69 (0.36, 1.32)	0.85 (0.48, 1.51)
Self-Rated Health (ref. = good)				
Moderate	0.72 (0.45, 1.13)	0.69 (0.45, 1.05)	0.61 (0.37, 1.01)	0.69 (0.44, 1.07)
Bad	0.68 (0.42, 1.10)	0.65 (0.41, 1.01)	0.60 (0.35, 1.01)	0.66 (0.42, 1.05)
Number of admissions (ref. = 1)	0.76 (0.50,1.17)	0.60 * (0.41,0.90)	0.68 (0.43,1.07)	0.76 (0.50,1.15)

Note: *** *p* < 0.001; ** *p* < 0.01; * *p* < 0.05. Listwise deletion method was used to manage missing value. The sample size for each model is smaller than original 1471. “ref” means “reference category.”
